# Metabolic profiling of idiopathic pulmonary fibrosis in a mouse model: implications for pathogenesis and biomarker discovery

**DOI:** 10.3389/fmed.2024.1410051

**Published:** 2024-08-07

**Authors:** Yu-zhu Zhang, Xiu-juan Jia, Wen-juan Xu, Xiao-qian Ding, Xiao-meng Wang, Xiao-sa Chi, Yi Hu, Xiao-hui Yang

**Affiliations:** ^1^Department of Geriatrics, The Affiliated Hospital of Qingdao University, Qingdao, China; ^2^Department of Respiratory and Critical Care Medicine, The Affiliated Hospital of Qingdao University, Qingdao, China

**Keywords:** pulmonary fibrosis, metabolites, machine learning, biomarkers, mice

## Abstract

**Background:**

Alterations in metabolites and metabolic pathways are thought to be important triggers of idiopathic pulmonary fibrosis (IPF), but our lack of a comprehensive understanding of this process has hampered the development of IPF-targeted drugs.

**Methods:**

To fully understand the metabolic profile of IPF, C57BL/6 J male mice were injected intratracheally with bleomycin so that it could be used to construct a mouse model of IPF, and lung tissues from 28-day and control IPF mice were analyzed by pathology and immunohistochemistry. In addition, serum metabolites from IPF mice were examined using LC-ESI-MS/MS, and the differential metabolites were analyzed for KEGG metabolic pathways and screened for biomarkers using machine learning algorithms.

**Results:**

In total, the levels of 1465 metabolites were detected, of which 104 metabolites were significantly altered after IPF formation. In IPF mouse serum, 52% of metabolite expression was downregulated, with lipids (e.g., GP, FA) and organic acids and their derivatives together accounting for more than 70% of the downregulated differentially expressed metabolites. In contrast, FA and oxidised lipids together accounted for 60% of the up-regulated differentially expressed metabolites. KEGG pathway enrichment analyses of differential metabolites were mainly enriched in the biosynthesis of unsaturated fatty acids, pentose phosphate pathway, and alanine, aspartate, and glutamate metabolism. Seven metabolites were screened by machine learning LASSO models and evaluated as ideal diagnostic tools by receiver operating characteristic curves (ROCs).

**Discussion:**

In conclusion, the serum metabolic disorders found to be associated with pulmonary fibrosis formation will help to deepen our understanding of the pathogenesis of pulmonary fibrosis.

## Introduction

Pulmonary fibrosis is a chronic progressive lung disease characterized by increased fibrosis (scarring) in the lung tissue leading to impaired lung function, often resulting in symptoms such as breathlessness, coughing, and chest pain, which can progressively worsen (1). Disease progression is unpredictable, with most patients appearing stable for the first few years, however, once an acute exacerbation occurs, symptoms can rapidly worsen over days or weeks. Most patients experience rapid deterioration of lung function within 3–5 years of diagnosis and experience respiratory failure or die after one or more acute exacerbations, which has been described as a “cancer that is not cancer” (2). The global incidence and prevalence of IPF are 0.09–1.30 and 0.33–4.51 per 10,000 persons, respectively (3).

However, the pathogenesis of pulmonary fibrosis is not fully understood, but in most cases, it is associated with chronic inflammation and abnormalities in damage repair processes (4). These abnormal responses are often closely linked to environmental factors, medication, immune system abnormalities, genetic factors, and infections (5). Prolonged exposure to harmful substances, dust, or pollutants in the environment, such as asbestos fibers, silica dust, organic solvents, sulfur dioxide, etc., may lead to pulmonary fibrosis. Certain medications and treatments such as antibiotics, chemotherapy drugs, and radiation therapy may cause pulmonary fibrosis. SARS-CoV-2, Human T-cell leukemia virus (HTLV), Human immunodeficiency virus (HIV), Cytomegalovirus (CMV), and Epstein–Barr virus (EBV) infections are associated with the development of pulmonary fibrosis (6).

The development of pulmonary fibrosis is accompanied by a range of metabolite alterations, including oxidative metabolites, inflammatory mediators and extracellular matrix metabolites, which may influence disease progression (7, 8). Abnormal accumulation and imbalance of these metabolites promote fibrotic tissue proliferation and deposition, ultimately leading to impaired lung function (9). During pulmonary fibrosis, oxidative metabolites such as free radicals and peroxides are produced and accumulate. These oxidative metabolites can trigger intracellular oxidative stress, leading to cellular damage and inflammatory responses that ultimately promote fibrous tissue proliferation and deposition (10). Inflammatory mediators such as cytokines, chemokines, and inflammatory cell-activating substances are released and accumulate in lung tissue, causing an inflammatory response (11). These inflammatory mediators can activate fibroblasts and other cell types, promoting the proliferation and deposition of fibrous tissue (12). Metabolites such as matrix metalloproteinases (MMPs) and tissue inhibitory metalloproteinases (TIMPs) are involved in the metabolism of the extracellular matrix (13). MMPs is capable of degrading matrix proteins, while TIMPs inhibit the activity of MMPs. In pulmonary fibrosis, the activity of MMPs increases while the level of TIMPs decreases, leading to excessive deposition of the extracellular matrix. In the pre-IPF phase, we identified more than one-third of the metabolites altered during IPF formation by constructing a mouse model of pulmonary fibrosis, and they were mainly enriched in pathways such as the metabolism of glycerol and glycerophospholipids (14). Metabolomic analysis of lung tissue from IPF mice by Jelena et al. showed an up-regulation of energy metabolites represented by glycolysis, tricarboxylic acid cycle, glutaminolysis, lactate production and fatty acid oxidation (15). It is important to note that the pathogenesis of pulmonary fibrosis is complex and may interact with multiple factors. Therefore, an accurate understanding of factors such as an individual’s medical history, environmental exposures, and family history is important for the prevention and treatment of pulmonary fibrosis.

Pulmonary fibrosis is currently incurable, and the main goal of treatment is to control the progression of the disease and prolong the patient’s life. Early diagnosis and intervention are what make it possible to stop further deterioration and maintain lung function at its current best (16). However, confirming the diagnosis of pulmonary fibrosis is challenging because the signs and symptoms may be similar to those of other lung diseases (e.g., chronic obstructive pulmonary disease, pulmonary thromboembolism, etc.) (17). Early symptoms of pulmonary fibrosis, such as dyspnoea, cough, and malaise, may also be similar to those of other lung diseases, and there may be no obvious specific signs on physical examination, making the diagnosis difficult. In addition, pulmonary fibrosis due to different etiologies may have different clinical features and courses, which adds to the complexity of the diagnosis.

The diagnosis of IPF is currently based on a combination of clinical symptoms, imaging tests, and pulmonary function tests, and no specific biomarkers have been identified for the diagnosis of pulmonary fibrosis (18). Nonetheless, some biomarkers may be associated with the development of pulmonary fibrosis and disease activity and can be used to assess the severity and prognosis of the disease. MUC5B is a gel-forming mucin secreted by bronchial glands and is a major component of respiratory mucus. Variations in the MUC5B gene have been associated with the pathogenesis of IPF disease and may be of value in assessing the prognosis of IPF disease (19). The alveolar and fine bronchial epithelial cells of IPF patients contained large amounts of MMP-7, whereas normal lung parenchyma contained minimal amounts. Serum and bronchoalveolar lavage MMP-7 levels were significantly higher in IPF patients than in healthy controls (20, 21). It should be noted that these biomarkers are not specific and cannot be used alone for the diagnosis of pulmonary fibrosis, thus making it difficult to meet clinical needs.

Therefore, simulating the pathological process of human pulmonary fibrosis disease by constructing a pulmonary fibrosis model can help to study the pathogenesis of the disease and find new therapeutic approaches (22). Bleomycin is a commonly used pulmonary fibrosis inducer, and the dosage and method can be precisely controlled to facilitate the regulation of the degree of fibrosis, and has been widely used in the study of IPF disease (23, 24). In this study, we constructed a mouse model of IPF treated with bleomycin from a metabolomics perspective and tried to find specific metabolites of IPF from a metabolite perspective for adjunctive diagnosis and disease assessment of pulmonary fibrosis.

## Materials and methods

The research was carried out in a randomized, controlled fashion. All experiments were authorized by the Qingdao University Affiliated Hospital Ethics Committee (Ethics No. QYFYW2LL26275), and all procedures were transported in conformity with the applicable laws and regulations. The research was performed adhering to the ARRIVE standards.

### Animals

We purchased 20 C57BL/6 J Specific Pathogen Free (SPF) healthy male mice weighing between 250 and 300 g and aged around 8 weeks from Beijing Vitality River Laboratory Animal Technology Co. and housed them in the Experimental Animal Centre of the Affiliated Hospital of Qingdao University. Mice were housed under a 12/12-h light/dark cycle and up to five animals per cage with a ventilation system, a temperature range of 22–25°C, and a relative humidity of 46–65%. Mice were fed *ad libitum* on standard chow and water. Experiments were started 1 week after the mice were acclimatized.

### Construction of the pulmonary fibrosis model

Twelve hours before the experiment, the mice were randomly assigned to two groups: a model group and a control group, each consisting of 10 mice. In the model group, mice were injected with 2.0 mg/kg of bleomycin into their trachea on day 0, while the control group received an equal injection of saline only. The mice were properly sedated with 1% pentobarbital sodium and then fixed supine on an operating table. The feeding, drinking, and body weight of the mice were recorded over the course of their 28-day confinement.

### Sample collection

Samples were fasted for 12 h before sample collection. After 28 days, lung tissue and blood were collected for subsequent testing. All mice were fixed on the operating table in the supine position and then euthanized by intraperitoneal injection of 150–200 mg/kg of sodium pentobarbital. After clipping the coat of the chest area of the mice and sterilizing the skin, a syringe needle was inserted vertically through the sternum to extract blood from the mice. Subsequently, the thoracic cavity of the mice was incised using scissors scissors and forceps, and the lung tissue was removed and preserved in a fixative solution.

### Pathological, immunohistochemical analysis

For mice after induction of pulmonary fibrosis, we routinely processed, paraffin-embedded, and sectioned lung tissues using previously described methods (14), followed by TGF-β1 and Masson staining, and positive intensities of the measured areas were read and analyzed (Appendix_method).

### UPLC-MS metabolome profiling

An LC-ESI-MS/MS system (UPLC, ExionLC AD; MS, QTRAP® System) was used to analyze blood samples for the extraction of metabolites using the previously mentioned approach (14). Software Analyst 1.6.3 was used to process the mass spectrometry data (Appendix_method).

### Data analysis

Orthogonal Partial Least Squares Discriminant Analysis (OPLS-DA) was employed to account for the homogeneity and reproducibility of the metabolite data. To find the different metabolites between normal and model mice, the R package *limma* was utilized. The statistically significantly changed metabolites were selected using the following criteria: *p*-value lower than 0.05 and absolute log2 Foldchange (log2 FC) larger than 0.32. R software (version 4.0.5) was used to perform the heatmap and hierarchical clustering analysis. Metabolomic datasets can be made less dimensional by using machine learning, which also increases prediction precision. The characteristic content is chosen using machine learning’s LASSO penalized logistic regression analysis (package *glmnet*), and the effectiveness of the material is then assessed using the receiver operating characteristic curve (ROC). The R software’s *ggplot2* tool for visualizing data.

Metaboanalyst^1^ was used to analyze metabolic pathways, and the MBRole 2.0 website^2^ was deployed to analyze metabolites. Histograms display the ultimate results.

## Results

### Establishing the mouse model of bleomycin-induced pulmonary fibrosis

**Figure 1:**
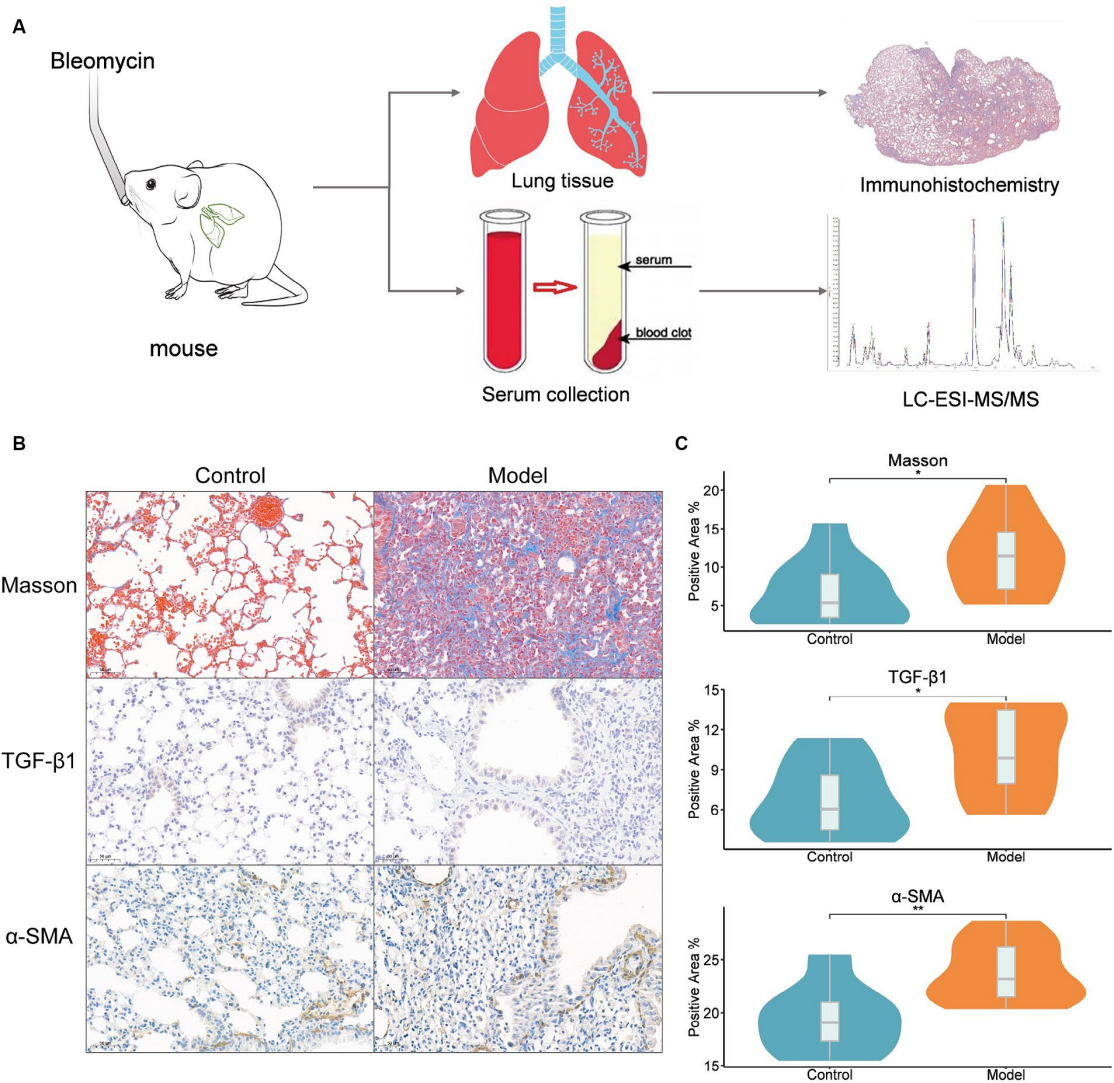
Bleomycin mouse models. **(A)** Study design, intratracheal instillation of bleomycin or saline (NaCl) was administered to anesthetized mice at day 0. Lung tissues and serum were collected from mice at 28 days of bleomycin induction and from control mice for histopathological and metabolomic assays; **(B)** Lung histopathology after a single dose of bleomycin treatment (Masson, TGF-β1, and ɑ-SMA staining). Representative Masson’s trichrome-stained (a and b), TGF-β1-stained (c and d), and ɑ-SMA-stained (e and f) lung tissue sections are shown. The mice were sacrificed at 28 days (b, d, and f) after bleomycin instillation. Except for the PBS control (a, c, and e), all panels showed lung sections from bleomycin-treated mice. The lungs were inflated with 10% buffered formaldehyde. Original magnification, 20X; **(C)** The results of Masson, TGF-β1 and ɑ-SMA immunohistochemistry. Positive area ratio (%), reflecting the proportion of the positive area. Statistical significance by two-tailed Student’s t-tests: ^∗^*p* < 0.05; ^∗∗^*p* < 0.01.

We injected mice with saltwater (NaCl) containing 2.0 mg/kg bleomycin into their trachea to completely comprehend the alterations in the metabolome throughout the development of lung fibrosis (Figure 1A). On day 28 (M28), lung tissues and serum were taken from 10 mice based on their lung fibrosis stage. Lung fibrosis is largely caused by TGF-β1, and immunohistochemistry of TGF-β1 in lung tissues (Figures 1B,C) revealed that the amount of TGF-β1 increased significantly (*p* < 0.05) as the degree of lung fibrosis increased. Furthermore, α-SMA acts as a cellular hallmark of fibrosis as well. The immunohistochemistry analysis of lung tissues revealed that α-SMA expression was considerably higher (*p* < 0.01) as well. The results of the Masson staining indicated a strong correlation between pulmonary fibrosis and an increase in collagen fibers in lung tissue.

### Metabolic alterations in the serum of fibrotic mice

**Figure 2:**
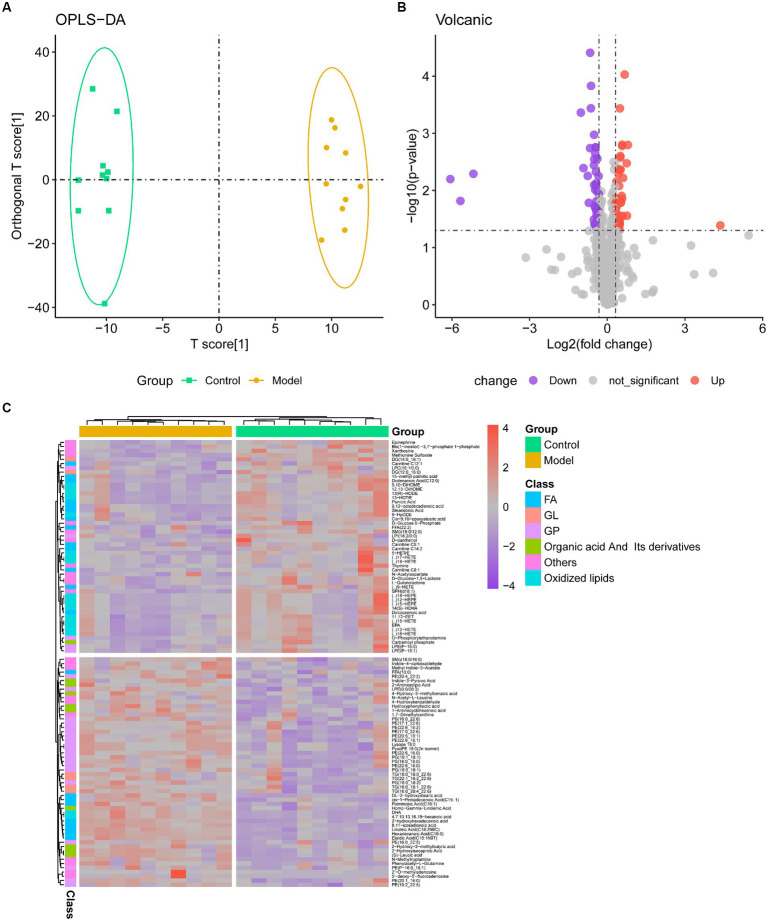
Metabolome profiling of serum. **(A)** The clustering analyses of orthogonal partial least-squares discriminant analysis (OPLS-DA) of Control and Model group (R2X = 0.349, R2Y = 0.990, Q2 = 0.527); **(B)** Volcano plot of metabolites, with purple representing downward trend, gray representing insignificance, and red representing upward trend; **(C)** Heatmap of the relative abundance of representative differential serum metabolites among Control and Model groups. GP, glycerophospholipids; FA, fatty acyls.

Using mass spectrometry (MS), metabolic profiles in serum from a mouse fibrosis model were obtained. Serum samples contained 1,465 metabolites in total, which were categorized into 19 classes, mostly fatty acyl (FA), GP, and GL (Supplementary Figure S1). OPLS-DA showed a clear trend of metabolite segregation showing differences between groups (Figure 2A). Using the *limma* package, we conducted a difference assessment between the model and control groups and discovered that 104 metabolites were expressed differently in the two groups (Figure 2B). Approximately 52 percent of the differential metabolites had down-regulated expression in IPF mouse serum, while the remaining metabolites had up-regulated expression (Figure 2C). Combined, lipids (such as GP and FA) and organic acid and its byproducts accounted for almost 70% of the differential metabolites that were down-regulated. There was a down-regulation of the metabolites with differential expression (Figure 2C). However, FA and oxidized lipids combined accounted for 60% of the differentially expressed metabolites that were up-regulated (Supplementary Table S1).

### Pathway enrichment analysis in mice with pulmonary fibrosis

**Figure 3:**
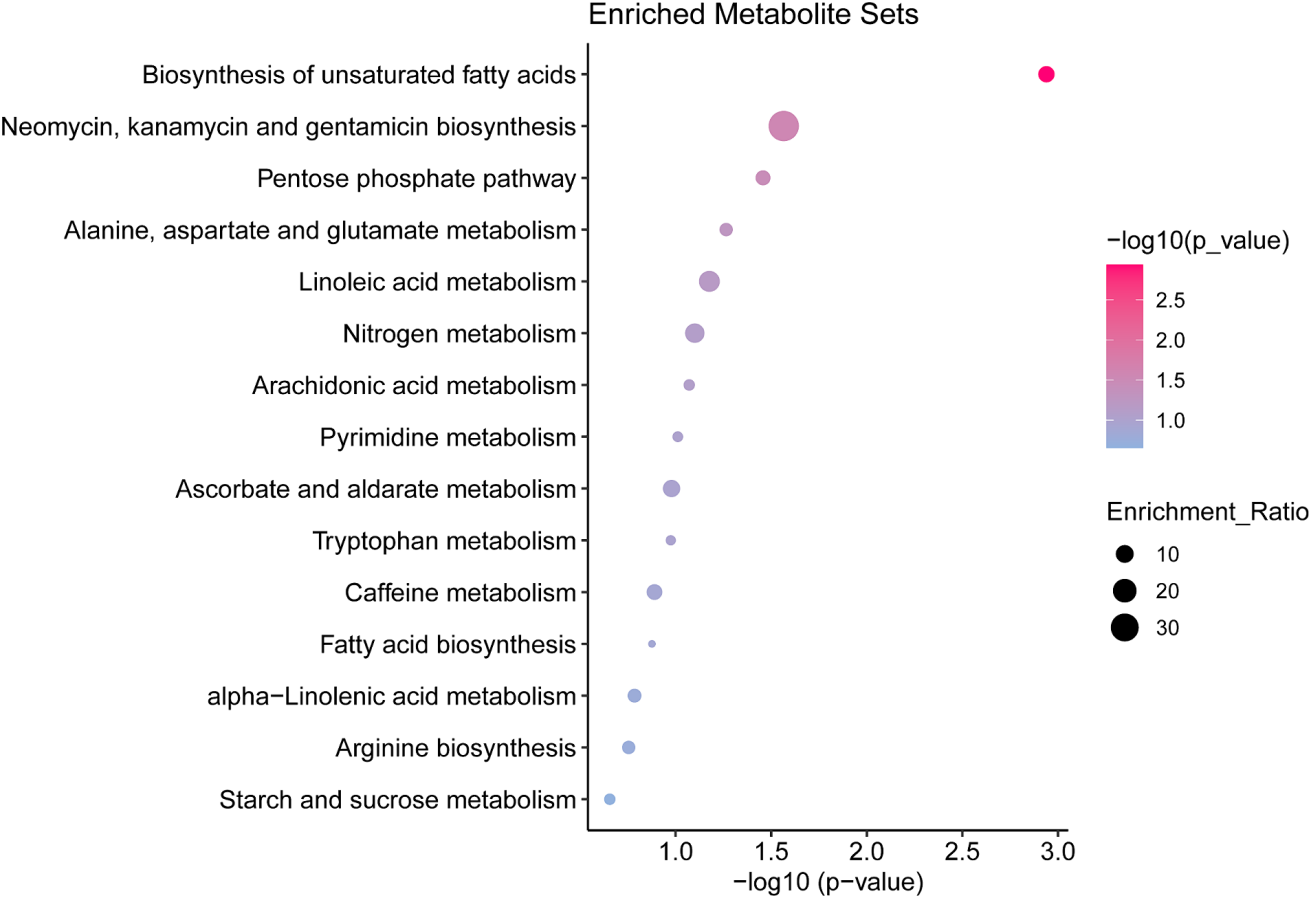
Pathway enrichment analysis of differential metabolites in the serum of mice with pulmonary fibrosis Results of differential metabolite enrichment analysis via the MBRole 2.0 website. The color of the dot represents the *p*-value, and the redder the bar, the more significant the pathway.

Enrichment and pathway analyses of metabolites that change during pulmonary fibrosis in mice were performed. Analysis of metabolites that differed between the IPF model and controls showed that mainly biosynthesis of unsaturated fatty acids, pentose phosphate pathway, and alanine, aspartate, and glutamate metabolism were enriched (Figure 3), which also hints at a strong link between the process of pulmonary fibrosis and energy metabolism and amino acid metabolism.

### Identification of potential metabolic biomarkers associated with lung fibrosis in mice

**Figure 4:**
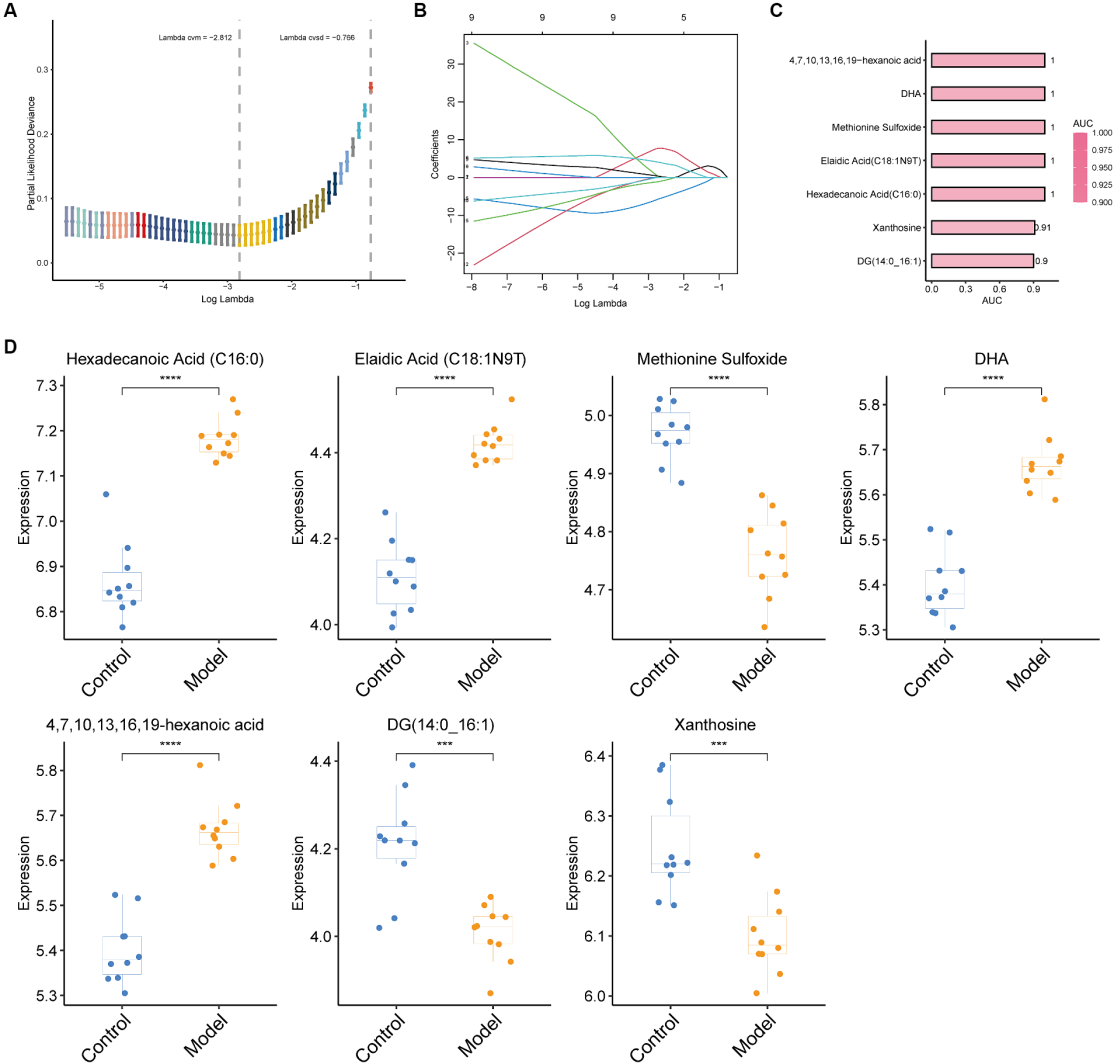
LASSO regression algorithm for screening biomarkers and evaluating their performance. **(A)** The optimal set of retinal parameters was selected by tenfold cross-validation and lambda. Min; **(B)** A coefficient profile plot was produced against the log(lambda) sequence; **(C)** Performance results of the seven metabolites screened as assessed by ROC curves. The larger the AUC, the redder the bar. **(D)** Box plots of the seven metabolites screened. Statistical significance by two-tailed Student’s *t*-tests: ^∗^*p* < 0.05; ^∗∗^*p* < 0.01; ^∗∗∗^*p* < 0.001; ^∗∗∗∗^*p* < 0.0001.

The aforementioned investigations demonstrated that mouse serum contains a wide variety of metabolites, which alter as the severity of pulmonary fibrosis in mice increases. Consequently, we created a LASSO model to choose the proper pulmonary fibrosis metabolic indicators. Seven metabolites were screened using this model, which was hexadecanoic acid (C16:0), elaidic acid (C18:1N9T), methionine sulfoxide, DHA, 4, 7, 10, 13, 16, 19-hexanoic acid, DG (14:0_16. 1) and xanthosine (Figures 4A,D). The areas under the ROC curves (AUC) for these seven metabolites were all more than 0.9 when we used ROC curves to evaluate their diagnostic usefulness. This suggests that the model (Figures 4B,C) is a perfect diagnostic tool for pulmonary fibrosis. The correlation analysis between immunohistochemistry indicators and these 7 metabolites shows that α-SMA, TGF- β1, and Masson are positively correlated with hexadecanoic acid (C16:0), elaidic acid (C18:1N9T), DHA, and 4, 7, 10, 13, 16, 19-hexanoic acid, while it is negatively correlated with methylene sulfoxide, DG (14:0_16 1) and xanthosine (Supplementary Figure S2).

## Discussion

The detailed demonstration of 1,465 metabolite alterations during lung fibrosis in mice was achieved by the use of metabolomics technologies. Of interest, 104 metabolites were significantly altered in the serum of bleomycin-treated mice, suggesting that lung fibrosis exerts an essential influence on the metabolism of mice.

The metabolism of pulmonary fibrosis is an emerging and worthwhile area of research, with current studies focusing on lipid metabolism, glucose metabolism, and collagen metabolism (25). TGF-β-stimulated IPF fibroblasts have been shown to exhibit higher transcript abundances of important rate-limiting enzymes encoding glycolytic enzymes, such as pyruvate kinase muscle isozyme M2 (PKM2), phosphofructokinase 1 (PFK1), and hexokinase 2 (HK2) (26, 27). Additionally, in fibroblast foci in lung epithelial cells, macrophages, and IPF lungs, as well as in experimental models of pulmonary fibrosis, there was an increase in 6-phosphofructo-2-kinase/fructose-2,6-bisphosphatase 3 (PFKFB3) (26, 28). Clonidine treatment attenuated the development of fibrosis and improved lung function in the bleomycin- and TGF-β-induced mouse model of pulmonary fibrosis, so targeting glycolysis for the treatment of fibrosis holds some promise. In addition, lactate excretion is a marker of enhanced glycolysis, and enhanced extracellular acidification and lactate production are thought to be key features of TGF-β-activated fibroblasts, which are critical for myofibroblast differentiation and collagen synthesis (29). Increased lactate in IPF lung tissues and bleomycin-induced fibrosis in a murine model of fibrosis may partly reflect increased lactate and glycolysis in myofibroblasts (25, 30). In the context of IPF, it has been proposed that abnormalities in lipid mediators (such as prostaglandins and lysophospholipids) may be the cause of the fibrotic response (31, 32). Less research has been done on the reprogramming of lipid production pathways to fibrosis. Following TGF-β stimulation, fatty acid synthase (FASN) mRNA and protein are elevated in human lung and murine fibroblasts as well as in bleomycin-induced mouse lungs (32). Our study additionally indicated modifications in lipid metabolism, glucose metabolism, and other pathways during pulmonary fibrosis, which may lead to a better understanding of the disease from a metabolic standpoint.

Important pathological characteristics of pulmonary fibrosis include modification of the extracellular matrix, interruption of collagen fiber metabolism, and sustained activation of fibroblasts/myofibroblasts. On day 28 after the mice were injected with bleomycin via the trache, we noticed aberrant extracellular matrix deposition and progressive lung tissue fibrosis. TGF-β1 concentrations in the tissue were also higher than expected, indicating a robust inflammatory response in the lung tissue that sped up the fibrosis process (33). Furthermore, there is a notable rise in the lung tissue’s α-SMA content. Among other things, α-SMA is produced by myofibroblasts, and the overaccumulation and sustained response of myofibroblasts at the site of injury is thought to underlie the continued progression of fibrosis in IPF and other diseases (34). Remarkably, although the administration of bleomycin has established an IPF mouse model that has been widely used in the study of IPF disease, we also need to use over genetic alteration or knockout of specific genes (e.g., knockout of Ifgr1/Rag2-related genes) to establish a pulmonary fibrosis model to gain insights into spontaneous pulmonary fibrosis, to study the pathogenesis of IPF and to search for new therapeutic approaches (35).

Owing to the relative difficulty in detecting IPF, efforts have been launched to identify a variety of non-invasive biomarkers, especially in serum, to help in the diagnosis of IPF. However, biomarkers like MUC5B and MMP-7 in bronchoalveolar lavage (BAL) fluid or serum are not specific and cannot be used alone for the diagnosis of pulmonary fibrosis, whereas metabolites in the blood have the potential to renew hope. Some studies have found that lysophosphatidylcholine (LysoPC) has the potential to be a biomarker in the serum of IPF patients (36). We used the random forest technique to examine 104 significantly changed metabolites and tested seven biomarkers to investigate the potential therapeutic utility of these altered metabolites. Four metabolites (DHA, 4, 7, 10, 13, 16, 19-hexanoic acid, Hexadecanoic Acid (C16:0), and Elaidic Acid (C18:1N9T)) showed a significant increase in serum levels as early as day 7 following bleomycin administration in mice, with sustained elevation observed at day 28 (Supplementary Figure S3). In contrast, the levels of the other three metabolites (Xanthosine, Methionine Sulfoxide, and DG (14;0_16:1)) exhibited a significant decrease by day 14 post-bleomycin administration and remained low at day 28 (14). Through ROC curve analysis, we were able to determine the very high diagnostic performance of these seven metabolites, indicating that they may be useful as biomarkers for the later diagnosis of pulmonary fibrosis and evaluation of the condition.

## Conclusion

In conclusion, we found significant changes in serum metabolism after IPF formation compared to control, including aberrant oscillations in pathways related to glucose and lipid metabolism. We are expecting a number of the metabolites from the serum that we analyzed to be useful biomarkers for clinical research.

## Data availability statement

The datasets presented in this study can be found in online repositories. The names of the repository/repositories and accession number(s) can be found at: https://db.cngb.org/search/project/CNP0005360/, CNP0005360.

## Ethics statement

The animal study was approved by Qingdao University Affiliated Hospital Ethics Committee. The study was conducted in accordance with the local legislation and institutional requirements.

## Author contributions

Y-zZ: Conceptualization, Data curation, Formal analysis, Investigation, Writing – original draft. X-jJ: Funding acquisition, Investigation, Methodology, Resources, Writing – original draft. W-jX: Formal analysis, Software, Supervision, Writing – original draft. X-qD: Formal analysis, Methodology, Project administration, Writing – original draft. X-mW: Validation, Visualization, Writing – review & editing. X-sC: Conceptualization, Resources, Supervision, Writing – review & editing. YH: Conceptualization, Investigation, Validation, Writing – original draft. X-hY: Funding acquisition, Resources, Validation, Visualization, Writing – review & editing.

## Footnotes

^1^ https://www.metaboanalyst.ca/MetaboAnalyst/

^2^ http://csbg.cnb.csic.es/mbrole2/
