# Supplementary material for: Metabolic profiling of idiopathic pulmonary fibrosis in a mouse model: implications for pathogenesis and biomarker discovery
Source: Front Med (Lausanne). 2024 Aug 7;11:1410051. doi: 10.3389/fmed.2024.1410051 (PMC11340507; doi:10.3389/fmed.2024.1410051)
Supplement: Supplementary file 2 [file Data_Sheet_1.docx]

# Appendix


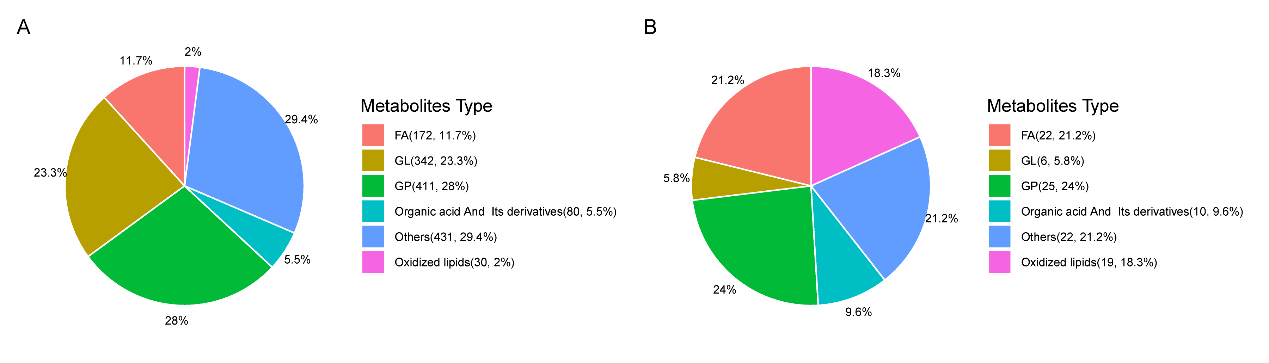


**Figure S1** **Pie chart of class distribution of all metabolites detected and differential metabolites**

(A) Pie chart of class distribution of all metabolites detected; (B) Pie chart of class distribution of differential metabolites.

**
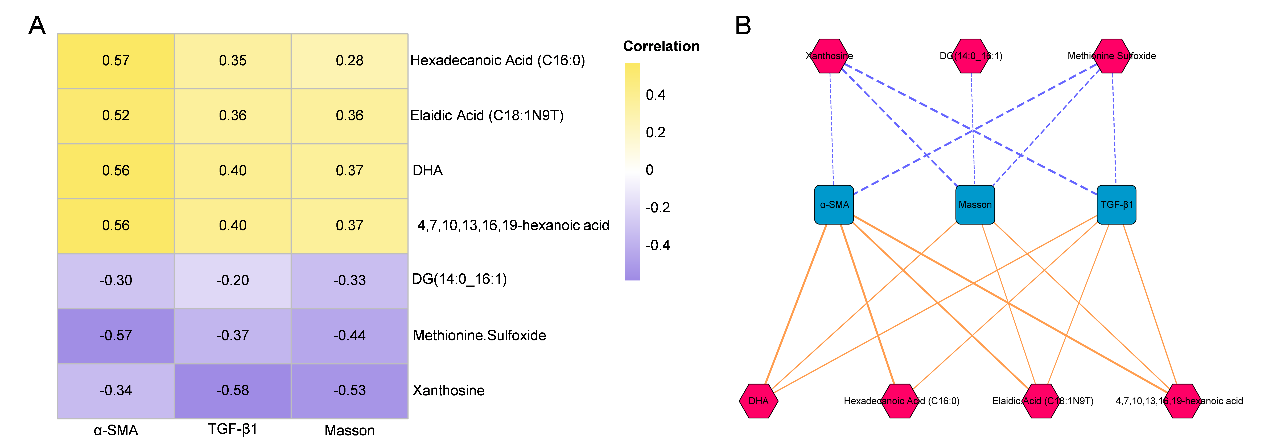
**

**Figure S2 Correlation between serum metabolites and immunohistochemistry of lung tissue**

(A) Heatmap based on spearman correlation test between serum metabolites and immunohistochemistry of lung tissue. Yellow indicates a positive correlation and purple is a negative correlation; the darker the color, the stronger the correlation. (B) Correlation network for serum metabolites and lung tissue immunohistochemistry. Red hexagons refer to metabolites and blue squares refer to different immunohistochemistry of lung tissue; solid lines indicate a positive correlation between the two and dashed lines indicate a negative correlation between the two; thicker lines indicate a stronger correlation and, conversely, a weaker correlation.


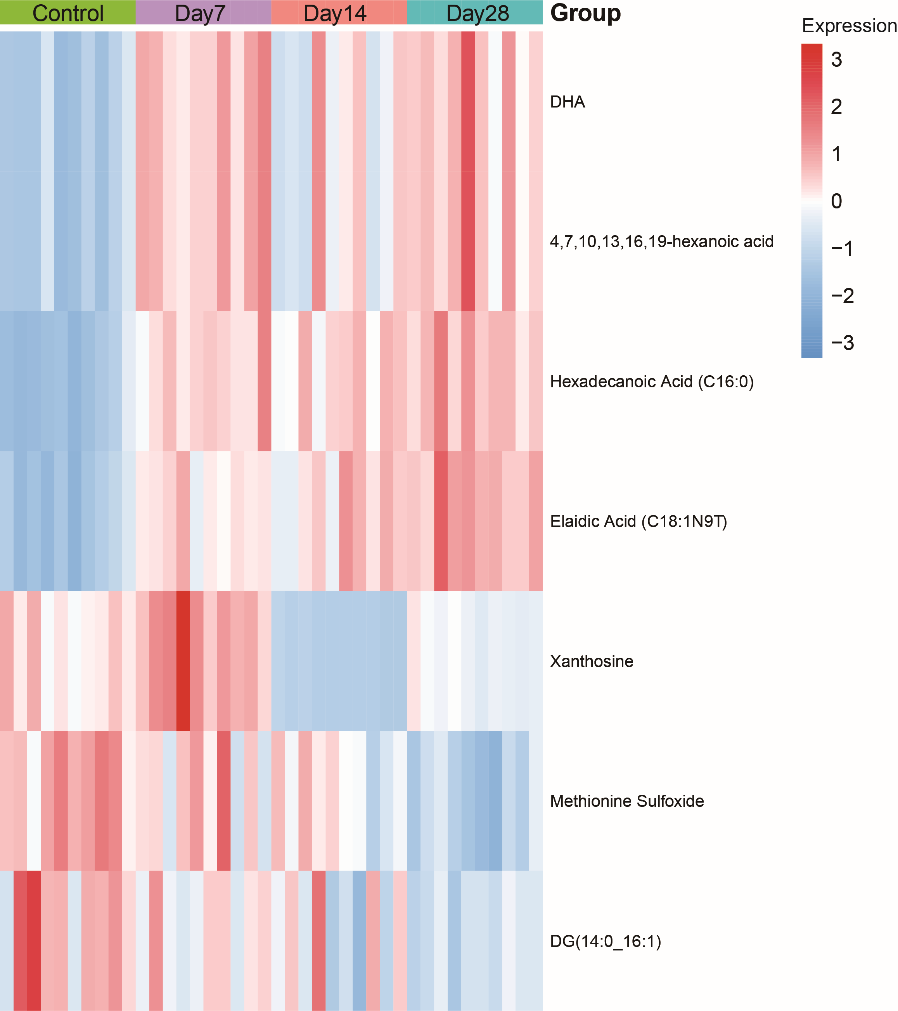


**Figure S3 Heatmap of the relative abundance of the screened seven metabolites at different time periods**

Red representing high expression and blue representing low expression. The 7- and 14-day metabolite data were obtained from the previous study, "Yang XH, Wang FF, Chi XS, et al. Disturbance of serum lipid metabolites and potential biomarkers in the Bleomycin model of pulmonary fibrosis in young mice. BMC Pulm Med 2022; 22(1):176".
