# Supplementary material for: Metabolic profiling of idiopathic pulmonary fibrosis in a mouse model: implications for pathogenesis and biomarker discovery
Source: Front Med (Lausanne). 2024 Aug 7;11:1410051. doi: 10.3389/fmed.2024.1410051 (PMC11340507; doi:10.3389/fmed.2024.1410051)
Supplement: Supplementary file 3 [file Data_Sheet_2.DOCX]

# METHODS

## Pathological, immunohistochemical analysis

The mice underwent a deep anesthesia with sodium pentobarbital and subsequently died from hemorrhage following an abdominal aortic incision. Following pulmonary fibrosis induction, the heart was perfused with PBS, and the left lobe received an injection of roughly 250 μl 4% paraformaldehyde. The tissue preserved with 4% paraformaldehyde was then processed according to standard procedure, embedded in paraffin, and sectioned. Screenshots depict representative slice areas. Two neighboring slices were stained with TGF-β1 and Masson, respectively. The stained slides were scanned with PANORAMIC DESK/MIDI/250/1000 (3DHISTECH, Hungary). The level of positivity of the measured areas was then read and analyzed using the software's image analysis system.

## UPLC-MS Metabolome Profiling

Metabolite extracts from serum samples were obtained by methanol-assisted protein precipitation, and LC-ESI-MS/MS analysis was performed on them (MS, QTRAP® System, https://sciex.com/; UPLC, ExionLC AD, https://sciex.com.cn/). Liquid phase separation was done using a Waters ACQUITY UPLC HSS T3 C18 column (1.8 m, 2.1 mm*100 mm). LIT and triple quadrupole (QQQ) scans were obtained utilizing a triple quadrupole linear ion trap mass spectrometer (QTRAP) equipped with Sciex's QTRAP® LC-MS/MS apparatus. A specific set of MRM alterations was seen depending on the metabolite elution at each interval. Software Analyst 1.6.3 was utilized for the processing of the mass spectrometry data. HMDB (http://www.hmdb.ca/), massbank (http://www.massbank.jp/), and metlin (http://metlin.scripps.edu/index.php) were the primary mass spectrometry public databases analyzed for the study of metabolite structure. Qualitative analysis was performed using retention lengths and mass-to-charge ratios of parent and daughter ions of test compounds from both internal and external databases. The quantitative analysis was performed using multiple reaction monitoring (MRM) techniques in triple quadrupole mass spectrometry. The detector measures the signal strength of the characteristic ions. MultiQuant is used to integrate and calibrate the chromatographic peaks. The peak area of each chromatographic peak indicates the pertinent chemical's relative concentration.
